# Pancreatic Plasmacytoma Presenting as Acute Pancreatitis: An Unusual Extramedullary Onset of Multiple Myeloma

**DOI:** 10.7759/cureus.85455

**Published:** 2025-06-06

**Authors:** Bakr Alhayek, Xiaowei Malone, Ariba Khan, Raja Gummalla, Ryan Brink

**Affiliations:** 1 Internal Medicine, AdventHealth Tampa, Tampa, USA

**Keywords:** acute pancreatitis, extramedullary disease, multiple myeloma, pancreatic mass, pancreatic plasmacytoma

## Abstract

Multiple myeloma (MM) typically manifests with bone pain, anemia, and hypercalcemia. Extramedullary disease (EMD), particularly pancreatic involvement, is rare. We report a 63-year-old woman presenting with acute pancreatitis secondary to a pancreatic plasmacytoma. Initial symptoms included epigastric pain, elevated lipase (1,389 U/L), and imaging revealing a 12.1 cm pancreatic mass. Biopsy confirmed lambda-restricted plasma cells (CD138+). Bone marrow biopsy showed 15% clonal plasma cells, meeting the diagnostic criteria for MM. EMD in MM signifies aggressive disease and poor prognosis.

## Introduction

Multiple myeloma (MM) is a hematologic malignancy characterized by the clonal proliferation of plasma cells and often initially presents with bone pain, hypercalcemia, and anemia. Extramedullary disease (EMD) of MM is uncommon, and pancreatic involvement is extremely rare [1]. Approximately 20% of patients present with plasma cell aggregates extending beyond the bone marrow at the time of myeloma diagnosis, and although pancreatic involvement by hematologic malignancies is intrinsically infrequent - with only about 150 cases of primary pancreatic lymphoma reported by 2011 - pancreatic plasma cell neoplasms are even more exceptional [1]. EMD arises when plasma cell subclones acquire bone marrow independence and infiltrate soft tissues. These subclones may harbor secondary genetic changes, such as TP53 mutations, t(4;14), and del(13). Alternatively, it has been hypothesized that aggressive, treatment-resistant clones emerge due to the elimination of sensitive cells by novel therapies; however, clinical evidence for this remains inconclusive [2]. Pancreatic involvement in MM poses a significant diagnostic challenge due to nonspecific symptoms such as abdominal pain, nausea, vomiting, and weight loss, which overlap with other pancreatic pathologies. Rarely, MM-associated pancreatic masses can present as acute pancreatitis, further complicating diagnosis [3]. Imaging modalities such as contrast-enhanced computed tomography (CT) and magnetic resonance imaging (MRI) often reveal nonspecific findings, necessitating histopathological analysis for definitive diagnosis.

Here, we present a case of a 63-year-old woman with MM, initially manifesting as pancreatic involvement, a rare and challenging extramedullary presentation.

## Case presentation

A 63-year-old Caucasian female with a past medical history significant for a 45-pack-year smoking history, diverticulitis status post resection, von Willebrand's disease, and gastroesophageal reflux disease (GERD) presented to the Emergency Department with the acute onset of severe epigastric pain radiating to the back, associated with nausea and vomiting for the past two weeks. The abdominal pain was constant and aggravated by eating. On examination, she was tachycardic, with tenderness to palpation in the epigastrium. Laboratory studies revealed an elevated lipase level of 1,389 U/L, hyponatremia, hypokalemia, acute kidney injury, and elevated liver transaminases (Table 1).

**Table 1:** Laboratory findings on admission BUN, Blood Urea Nitrogen; eGFR, Estimated Glomerular Filtration Rate; AST, Aspartate Aminotransferase; ALT, Alanine Aminotransferase; ALP, Alkaline Phosphatase; LDH, Lactate Dehydrogenase; WBC, White Blood Cell; A/G Ratio, Albumin/Globulin Ratio; CA 19‑9, Carbohydrate Antigen 19‑9; CA‑125, Cancer Antigen 125; IgG, Immunoglobulin G; IgM, Immunoglobulin M; IgA, Immunoglobulin A

Test	Result	Reference Range
Lipase	1389 U/L	0-60 U/L
Sodium	132 mmol/L	135-145
Potassium	3.2 mmol/L	3.5-5.1
BUN	32 mg/dL	7-18
Creatinine	4.50 mg/dL	0.6-1.3
eGFR	10.4 mL/min/1.73 m²	>60
Albumin	2.8 g/dL	3.5-5.0
Total protein	10.2 g/dL	6.0-8.3
Globulin (calc)	7.4 g/dL	2.3-3.5
A/G ratio	0.27	1.0-2.5
AST	84 U/L	0-40
ALT	92 U/L	0-41
ALP	570 U/L	44-147
Total bilirubin	1.5 mg/dL	0.2-1.2
LDH	351 U/L	135-225
Hemoglobin	8.6 g/dL	12-16
WBC	4.62 × 10³/µL	4-10
CA 19‑9	152 U/mL	0-37
CA‑125	58 U/mL	0-35
IgG	6207 mg/dL	700-1600
IgM	7 mg/dL	40-230
IgA	13 mg/dL	70-400

**Figure 1:**
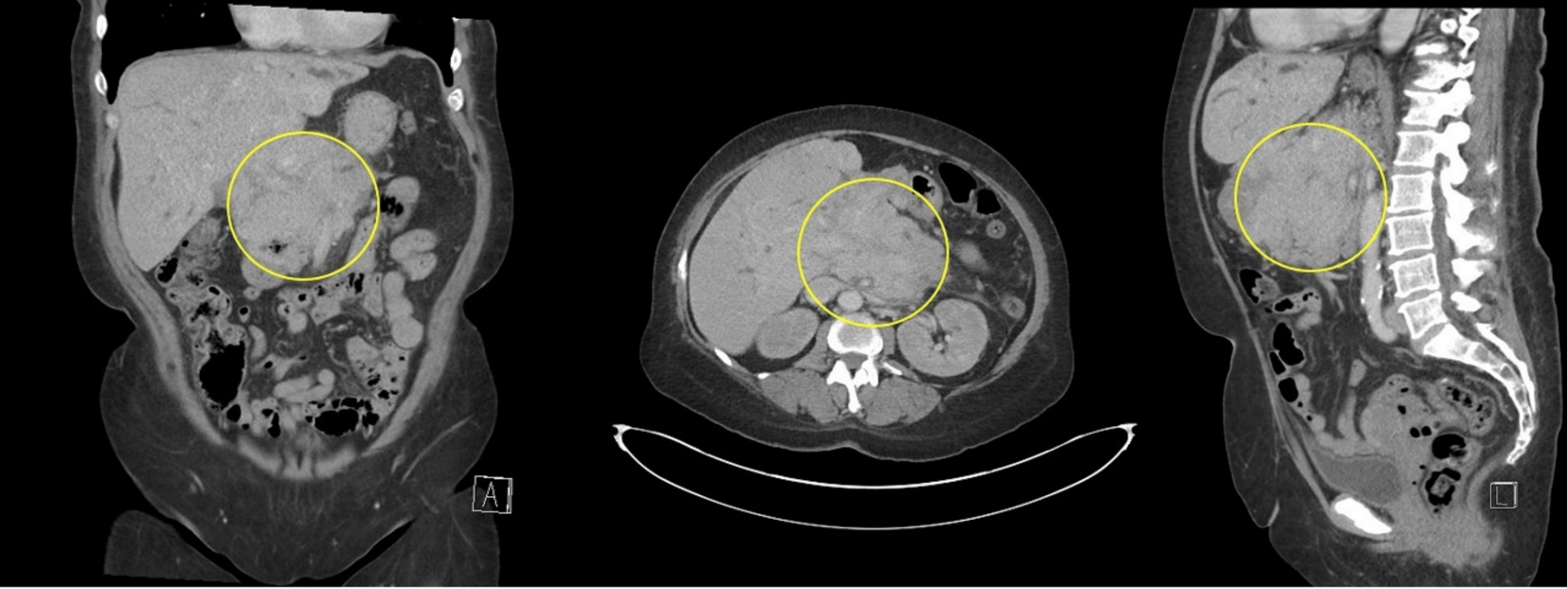
Coronal (left), axial (center), and sagittal (right) contrast-enhanced CT of the abdomen Coronal (left), axial (center), and sagittal (right) contrast-enhanced CT images of the abdomen show a large, lobulated soft-tissue mass (circled), replacing the majority of the pancreatic head and body. The lesion measures approximately 12.1 × 9.0 × 7.9 cm, encases the SMA and portal vein, and extends toward the region of the gallbladder fossa. Note the minimal prominence of the pancreatic duct within the mass.​ CT, Computed Tomography; SMA, Superior Mesenteric Artery

**Figure 2:**
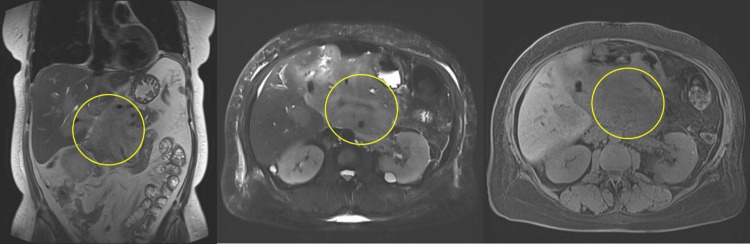
Coronal (left), axial (center), and axial (right) magnetic resonance images (MRCP protocol, no IV contrast) of the abdomen Coronal (left), axial (center), and axial (right) MR images (MRCP protocol, no IV contrast) demonstrate a large lesion (circled) arising from the porta hepatis and encompassing the pancreatic head. The mass extends along the posterior wall of the stomach and involves the first and second portions of the duodenum. Note the dilated pancreatic duct, which remains visible in its entirety, despite the extensive involvement by the lesion.​ MRCP, Magnetic Resonance Cholangiopancreatography

Based on these findings, acute pancreatitis was suspected, and intravenous fluids were initiated. However, a contrast-enhanced CT of the abdomen and pelvis revealed a large, lobular pancreatic head mass measuring 12.1 × 9.0 × 7.9 cm, encasing the superior mesenteric artery and portal vein, and contacting the liver at the porta hepatis (Figure 1). On the MRCP (magnetic resonance cholangiopancreatography) protocol MRI (without IV contrast), an approximately 18 cm lesion arises from the porta hepatis and extends into the pancreatic head, hepatic hilum, and duodenum (Figure 2). The pancreatic duct is dilated, yet remains intact.

**Figure 3:**
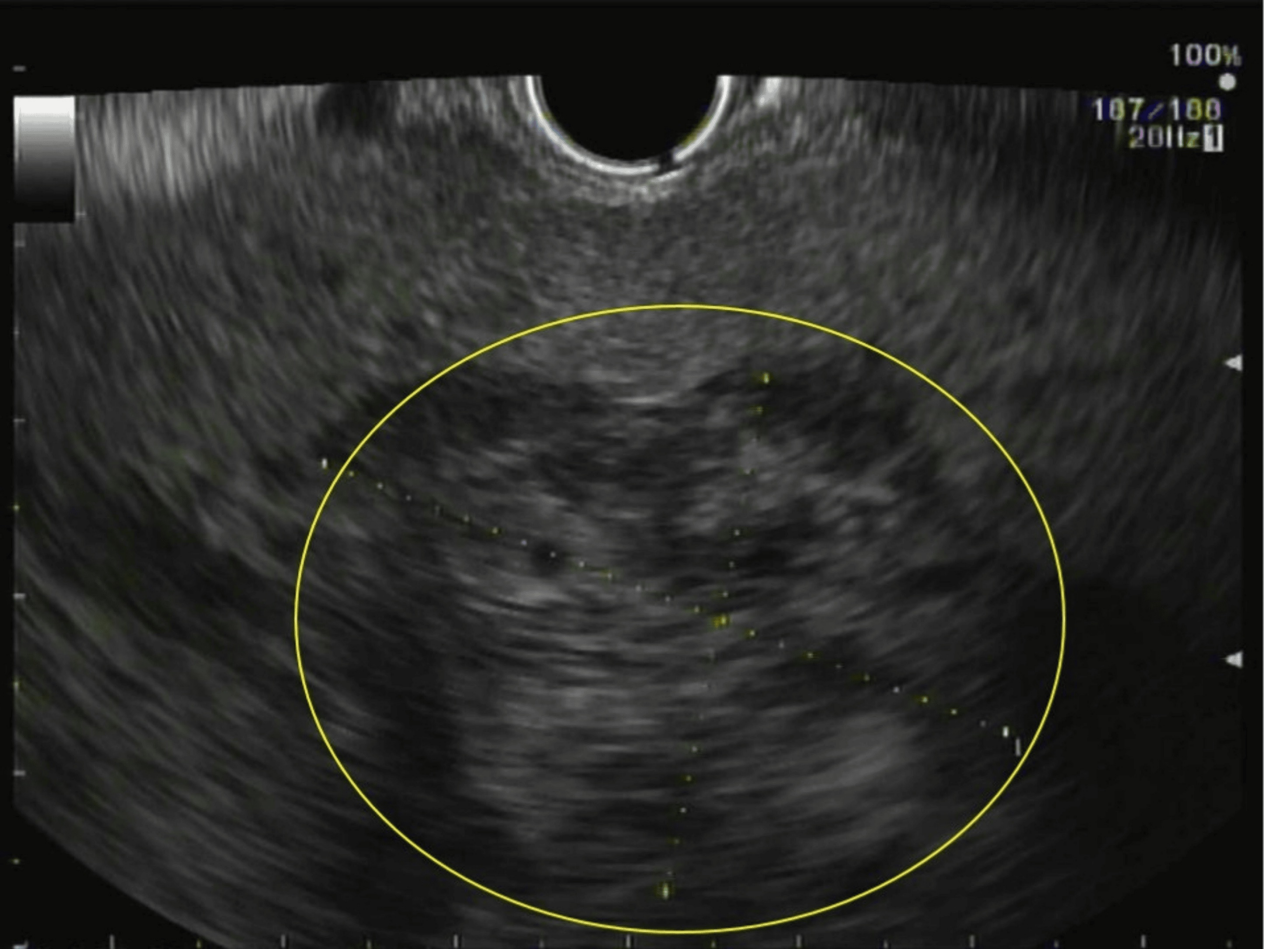
EUS image EUS image obtained with a therapeutic curvilinear echoendoscope demonstrates a large, heterogeneous mass (circled) around the pancreatic head (dimensions 6.9 × 4.1 cm), extending into the hepatic hilum as a contiguous 4.25 × 2.97 cm lesion. The mass involves multiple blood vessels (including the splenic and common hepatic arteries), encases the extrahepatic bile duct, and compresses the main pancreatic duct (measuring approximately 2.7 mm).​ EUS, Endoscopic Ultrasound

**Figure 4:**
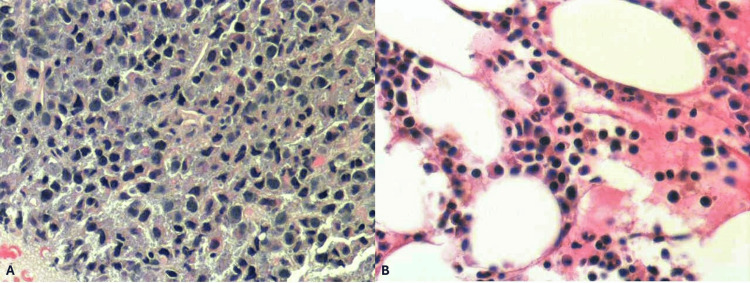
H&E‐stained sections comparing the pancreatic and bone marrow involvement by the plasma cell neoplasm (A) Pancreatic biopsy showing sheets of atypical plasma cells replacing normal exocrine tissue. These cells exhibit eccentric nuclei, “clock-face” chromatin, and perinuclear clearing (“hof”). (B) Bone marrow biopsy demonstrating numerous similar neoplastic plasma cells interspersed between large adipocytes (clear vacuoles), confirming concurrent medullary involvement.​ H&E, Hematoxylin and Eosin

**Figure 5:**
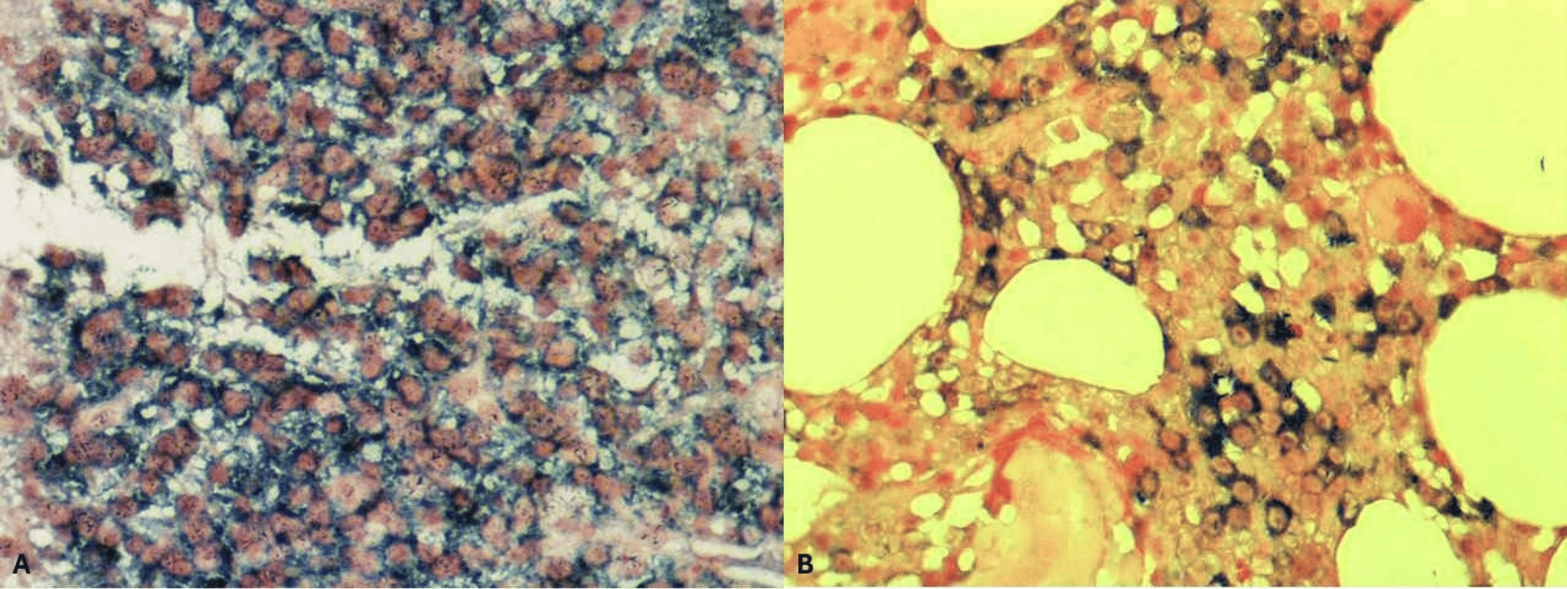
Immunostains highlighting lambda light-chain restriction in the neoplastic plasma cells of both the pancreas (A) and bone marrow (B) The brown cytoplasmic staining underscores a monoclonal (lambda-restricted) population, correlating with the final diagnosis of a systemic plasma cell neoplasm involving both sites.​

**Figure 6:**
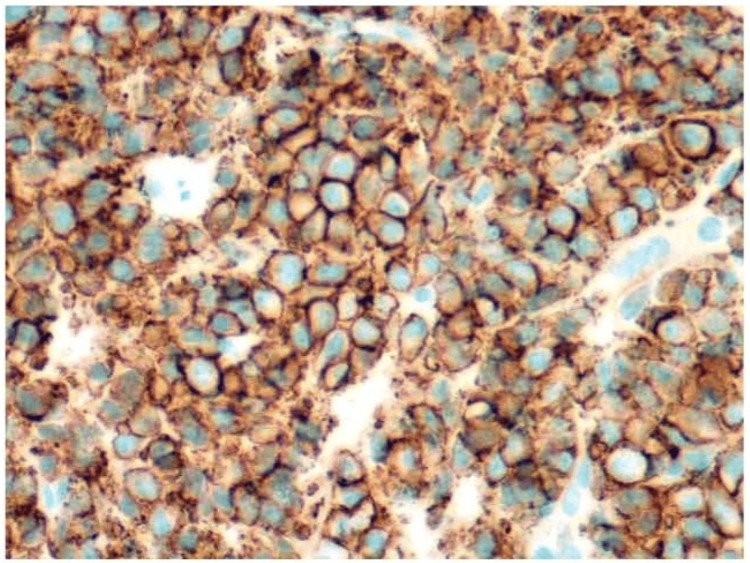
Immunohistochemical stain for CD138 Immunohistochemical stain for CD138 demonstrates robust membranous staining in the neoplastic plasma cells, confirming their plasmacytic differentiation. The uniform brown rim around each cell underscores the cohesive, clonal nature of this plasma cell population.​

Subsequently, an endoscopic ultrasound (EUS)-guided fine-needle biopsy of the mass was performed (Figure 3). Immunoglobulin (Ig) studies confirmed significantly elevated immunoglobulin G (IgG), with minimal immunoglobulin M (IgM) and immunoglobulin A (IgA) (Table 1). Further evaluation included a bone marrow biopsy, an MRI of the spine and pelvis, and a skeletal survey. The skeletal survey demonstrated calvarial lytic lesions. Bone marrow aspirate and biopsy (Figures 4B-5B) of the left iliac crest showed a monoclonal lambda plasma cell population comprising approximately 15% of the cellular marrow by in situ hybridization (ISH). Histopathology was consistent with pancreatic involvement by a plasma cell neoplasm (Figure 4A), with immunohistochemistry positive for CD138 (Figure 6). The ISH study demonstrated lambda light-chain restriction.

MRI revealed abnormal marrow signal within the spine, and multiple high T2 signal nodules within the intramedullary space of the pelvis. The osseous involvement supports that the pancreatic mass likely originated from plasma cell myeloma, instead of a primary plasmacytoma. The presence of >10% clonal bone marrow plasma cells, extramedullary plasmacytoma in the pancreas, and anemia was supportive of a new MM diagnosis, based on the International Myeloma Working Group (IMWG) diagnostic criteria [4]​. 

Induction therapy with bortezomib, lenalidomide, and dexamethasone (VRd) commenced on hospital day 10. By week 4, the serum M‑spike had fallen by more than 50%, and the lipase had normalized. A follow‑up CT at week 8 demonstrated an approximately 40% reduction in tumor volume, denoting a very good partial response. At that point, the patient was transferred to a regional myeloma center for stem‑cell mobilization and planned autologous transplantation. Long‑term outcome data were not available.

## Discussion

Pancreatic infiltration by MM remains exceptionally uncommon. EMD accompanies 7%-20% of newly diagnosed cases, yet the pancreas accounts for <1% of reported sites [5]. When EMD develops, overall survival falls dramatically - from roughly five years in marrow-confined MM to 12-24 months in contemporary series [6]. This adverse prognosis reflects biologic hallmarks that facilitate egress from the marrow microenvironment: down-regulation of adhesion molecules such as CD56 and CD138, overexpression of chemokine receptors (CCR1/2 and CXCR4), and enrichment for high-risk cytogenetic lesions (del(17p), 1q21 gain, and t(4;14)) [7]. Our patient exhibited del(13q14) and 1q21 gain, findings that align with this high-risk signature.

A systematic PubMed review (1950-2016) of 63 pancreatic plasmacytoma reports found predominantly middle-aged men (median age: 58.5 years) presenting with obstructive jaundice (70%) or pain (36%). Most cases were confirmed by EUS-guided fine-needle aspiration (EUS-FNA) (33%) or CT-guided fine-needle aspiration (CT-FNA) (21%). Management centered on chemotherapy (56%) and/or radiotherapy (RT) (52%), with surgery in 32% and biliary stenting in nearly half of the jaundiced patients - achieving universal objective tumor regression. Acute pancreatitis - our index presentation - was noted in only two cases, and survival outcomes were inconsistently reported [8].

Initial care followed standard pancreatitis protocols (hydration, analgesia, and bowel rest), while diagnostic studies confirmed myeloma-related disease. For solitary pancreatic plasmacytoma, definitive RT remains the gold standard [9]; in the 50-case series by Lopes da Silva, 40-50 Gy over four to six weeks achieved ~95% local control and 60% five-year survival [1]. Because our patient had concurrent marrow disease and high-risk cytogenetics, systemic therapy was prioritized.

Guided by IMWG 2023 recommendations, we started VRd on day 10 [10]. The M-spike fell >90% after four cycles, and stem-cell collection was planned after Cycle 6 for autologous transplantation, an approach linked to longer survival in pancreatic EMD [11]. Carfilzomib- or daratumumab-based triplets are reasonable substitutes when VRd is contraindicated, though prospective data are sparse [12]. Surgery is limited to bypass or hemostasis when other modalities fail, as cytoreductive pancreatectomy rarely controls systemic plasmacytoma [1].

Looking forward, novel immunotherapies - daratumumab-containing quadruplets in the frontline setting, and chimeric antigen-receptor T-cell constructs or bispecific T-cell engagers in relapsed disease - are reshaping the EMD landscape [13].

Clinicians should keep MM in the differential diagnosis of idiopathic pancreatitis accompanied by a discrete mass. Early ordering of serum protein electrophoresis, urine protein studies, and free-light-chain assays, followed by EUS-guided core biopsy, can prevent diagnostic delay. Prompt initiation of proteasome inhibitor-based induction, timely evaluation for autologous stem cell transplantation (ASCT), and judicious use of RT constitute the present standard of care, while emerging cellular therapies hold promise for refractory disease.

Limitations

This is a single-patient report with short follow-up and incomplete baseline prognostic data (e.g., β-2 microglobulin and fluorodeoxyglucose positron emission tomography), which restricts generalizability; larger series are needed to validate these observations.

## Conclusions

When pancreatitis has no clear cause and imaging shows a pancreatic mass, think plasmacytoma early. Pancreatic plasmacytoma marks extramedullary myeloma - an aggressive form that requires systemic therapy (proteasome-inhibitor/immunomodulatory drug (IMiD)-based chemotherapy ± transplant), not surgical resection. EUS biopsy, paired with cytogenetics and imaging, secures the diagnosis, and a multidisciplinary plan of prompt induction, selective local palliation, and vigilant follow-up offers the best chance of control.

## References

[1] Lopes da Silva R (2012). Pancreatic involvement by plasma cell neoplasms. J Gastrointest Cancer.

[2] Sevcikova S, Minarik J, Stork M, Jelinek T, Pour L, Hajek R (2019). Extramedullary disease in multiple myeloma - controversies and future directions. Blood Rev.

[3] Paydas S (2019). Pancreatic plasmacytoma: a rare but important entity for gastroenterologists, oncologists and hematologists. J Oncol Sci.

[4] Rajkumar SV (2016). Updated diagnostic criteria and staging system for multiple myeloma. Am Soc Clin Oncol Educ Book.

[5] Bladé J, Beksac M, Caers J (2022). Extramedullary disease in multiple myeloma: a systematic literature review. Blood Cancer J.

[6] Usmani SZ, Heuck C, Mitchell A (2012). Extramedullary disease portends poor prognosis in multiple myeloma and is over-represented in high-risk disease even in the era of novel agents. Haematologica.

[7] Fernández de Larrea C, Kyle RA, Durie BG (2013). Plasma cell leukemia: consensus statement on diagnostic requirements, response criteria and treatment recommendations by the International Myeloma Working Group. Leukemia.

[8] Williet N, Kassir R, Cuilleron M (2017). Difficult endoscopic diagnosis of a pancreatic plasmacytoma: case report and review of literature. World J Clin Oncol.

[9] Elsayad K, Oertel M, König L (2020). Maximizing the clinical benefit of radiotherapy in solitary plasmacytoma: an international multicenter analysis. Cancers (Basel).

[10] Sabry W, Wu Y, Kodad SG (2022). Bortezomib, lenalidomide and dexamethasone combination induced complete remission in relapsed/refractory plasmablastic lymphoma: case report of a potential novel treatment approach. Curr Oncol.

[11] Jantunen E, Koivunen E, Putkonen M, Siitonen T, Juvonen E, Nousiainen T (2005). Autologous stem cell transplantation in patients with high-risk plasmacytoma. Eur J Haematol.

[12] Paner A, Okwuosa TM, Richardson KJ, Libby EN (2018). Triplet therapies - the new standard of care for multiple myeloma: how to manage common toxicities. Expert Rev Hematol.

[13] Marcon C, Simeon V, Deias P (2022). Experts' consensus on the definition and management of high risk multiple myeloma. Front Oncol.

